# Sample sizes for cancer trials where Health Related Quality of Life is the primary outcome

**DOI:** 10.1054/bjoc.2000.1383

**Published:** 2000-09-04

**Authors:** S A Julious, M J Campbell, S J Walker, S L George, D Machin

**Affiliations:** Clinical Pharmacology Statistics, SmithKline Beecham, New Frontiers Science Park (South), Third Avenue, Harlow, Essex CM19 5AW; Institute of General Practice & Primary Care, University of Sheffield, Community Sciences Centre, Northern General Hospital, Herries Road, Sheffield, S5 7AU; Department of Medical Statistics & Computing, University of Southampton, Level B South Academic Block, Southampton General Hospital, Tremona Road, Southampton, SO16 6YD; Health Care Research Unit, Level B South Academic Block, University of Southampton, Southampton General Hospital, Tremona Road, Southampton, SO16 6YD; Institute of General Practice & Primary Care, University of Sheffield, Community Sciences Centre, Northern General Hospital, Herries Road, Sheffield, S5 7AU, UK

**Keywords:** Health Related Quality of Life, cancer, samples size, effect size

## Abstract

Health Related Quality of Life (HRQoL) instruments are increasingly important in evaluating health care, especially in cancer trials. When planning a trial, one essential step is the calculation of a sample size, which will allow a reasonable chance (power) of detecting a pre-specified difference (effect size) at a given level of statistical significance. It is almost mandatory to include this calculation in research protocols. Many researchers quote means and standard deviations to determine effect sizes, and assume the data will have a Normal distribution to calculate their required sample size. We have investigated the distribution of scores for two commonly used HRQoL instruments completed by lung cancer patients, and have established that scores do not have the Normal distribution form. We demonstrate that an assumption of Normality can lead to unrealistically sized studies. Our recommendation is to use a technique that is based on the fact that the HRQoL data are ordinal and makes minimal but realistic assumptions. © 2000 Cancer Research Campaign


British Journal of Cancer (2000) 83(7), 959–963
© 2000 Cancer Research Campaign
doi: 10.1054/ bjoc.2000.1383, available online at http://www.idealibrary.com on IDEAL®

### Sample sizes for cancer trials where Health Related Quality of Life is the primary outcome

**SA Julious[a], MJ Campbell[b], SJ Walker[c], SL George[d] and D Machin[e]**

[a]Clinical Pharmacology Statistics, SmithKline Beecham, New Frontiers Science Park (South), Third Avenue, Harlow, Essex, CM19 5AW; [b]Institute of General Practice & Primary Care, University of Sheffield, Community Sciences Centre, Northern General Hospital, Herries Road, Sheffield, S5 7AU; [c]Department of Medical Statistics & Computing, University of Southampton, Level B South Academic Block, Southampton General Hospital, Tremona Road, Southampton, SO16 6YD; [d]University of Southampton, Health Care Research Unit, Level B South Academic Block, Southampton General Hospital, Tremona Road, Southampton, SO16 6YD; [e]Institute of General Practice & Primary Care, University of Sheffield, Community Sciences Centre, Northern General Hospital, Herries Road, Sheffield, S5 7AU, UK

**Summary** Health Related Quality of Life (HRQoL) instruments are increasingly important in evaluating health care, especially in cancer trials. When planning a trial, one essential step is the calculation of a sample size, which will allow a reasonable chance (power) of detecting a pre-specified difference (effect size) at a given level of statistical significance. It is almost mandatory to include this calculation in research protocols. Many researchers quote means and standard deviations to determine effect sizes, and assume the data will have a Normal distribution to calculate their required sample size. We have investigated the distribution of scores for two commonly used HRQoL instruments completed by lung cancer patients, and have established that scores do not have the Normal distribution form. We demonstrate that an assumption of Normality can lead to unrealistically sized studies. Our recommendation is to use a technique that is based on the fact that the HRQoL data are ordinal and makes minimal but realistic assumptions. © 2000 Cancer Research Campaign

**Keywords:** Health Related Quality of Life; cancer; samples size; effect size

Health Related Quality of Life (HRQoL) has become an important endpoint in cancer clinical trials (de Haes and van Knippenberg, 1985; Fayers and Machin, 1995) and a general review, including a survey of which measures are used in practice, has been made by Campbell et al (2000). HRQoL is particularly valuable in assessing palliative treatments in situations where the size of any survival advantage for a new treatment is, at most, modest. Thus, there is a need to quantify the benefit of certain medical interventions in terms of a difference in HRQoL score rather than by improvement in survival alone.

The sample size required for a clinical trial is critically dependent on the pre-specified Type I error rate $\alpha$, the pre-specified Type II error rate $\beta$ (which gives the power, defined as $1-\beta$), and the anticipated clinically meaningful difference in HRQoL score (effect size), which are all interlinked (Machin et al, 1997). Since HRQoL scales form ordered categories by definition, sometimes they are far from appearing Normal in form and neither can they be transformed into being approximately so. Often these measures are subject to ‘floor’ or ‘ceiling’ effects in which the lowest or highest category predominates.

In this paper, using data on HRQoL outcome scores from lung cancer patients in a clinical trial, we demonstrate how the asymmetric distribution of the measure has an important impact on the sample size calculations. We provide a comparison of two methods of estimating sample sizes, one under the assumption of normality and one that is distribution-free. We illustrate how markedly different sample sizes are obtained and that one could either under or over recruit patients to a trial depending on the direction of the treatment effect. Also highlighted is how one solution often applied to calculate sample sizes for non-normal data, which is to dichotomize around a known cut-point, may substantially overestimate the required sample size.

*Received 18 January 2000*
*Revised 9 June 2000*
*Accepted 13 June 2000*

*Correspondence to:* Steven A. Julious

### MATERIALS AND METHODS

### The data

The data in this paper are taken from a randomized parallel group controlled trial of a standard treatment against a less intensive treatment in 310 patients with small-cell lung cancer and poor prognosis (Medical Research Council Lung Cancer Working Party, 1996). The standard treatment (*A*) consisted of a four-drug regime (etoposide, cyclophosphamide, methotrexate and vincristine) while the new less intensive treatment (*B*) under investigation contained just two of these compounds (etoposide and vincristine). The two treatment schedules were the same, comprising three cycles of chemotherapy at the same dosage. Each cycle was given on three consecutive days at three-week intervals.

### The HRQoL questionnaires

The two HRQoL questionnaires used in this trial were the Hospital Anxiety and Depression Scale (HADS) (Zigmond and Snaith, 1983) and the Rotterdam Symptom Checklist (RSCL) (de Haes et al, 1990)

The HADS provides scores in the range 0–21 in two dimensions: anxiety and depression. It is a self-rating questionnaire completed while patients wait to see a doctor and was developed for use in a general outpatient setting. Moorey et al (1991) reported that HADS is a useful instrument for measuring these dimensions in cancer patients. The HADS has three clinically predefined categories for each dimension: a total score 0–7 is defined as a ‘normal’, 8–10 as a ‘borderline-case’ and 11–21 as a ‘case’ suggesting significant anxiety or depression.

The RSCL has two main scales, physical symptom distress and psychological distress, in addition to the scales for activity and overall evaluation. It was developed to measure the symptoms of cancer patients participating in clinical research. Patients indicate how much they have experienced particular symptoms over the last week. The RSCL psychological dimension, for example, has scores ranging from 0 to 24, where high scores constitute psychological distress. It has two clinically pre-defined categories where a total score of 0–10 is considered a ‘non-case’ and 11–24 is a ‘case’ considered to constitute psychological distress.

In the trial setting both HRQoL questionnaires were completed together and the 310 patients’ baseline scores prior to randomization are used in this paper for expository purposes.

### Sample size methodology

In the following, $N$ is the total number of patients required in the trial for a pre-specified Type I error rate, α, and power, 1-β, where power is the probability of rejecting the null hypothesis given that it is false. $Z_{1-\alpha/2}$ and $Z_{1-\beta}$ are the appropriate values from the standard Normal distribution for the 100 (1-α/2)% and 100 (1-β)% percentiles respectively. Maximum power for a fixed number of patients is achieved by dividing $N$ into equal numbers of subjects in each treatment group.

### *Normal distribution method*

Assuming that the data have a Normal distribution, then the sample size required to compare two means $\mu_A$ and $\mu_B$, for a given effect size $\delta = \mu_A - \mu_B$ is given by Machin et al (1997) as:

$$N = \frac{4(Z_{1-\alpha/2} + Z_{1-\beta})^2}{d^2} + \frac{Z^2_{1-\alpha/2}}{2}. \qquad (1)$$

Here, the standardized difference is $d = \delta/\sigma$, where σ is the true standard deviation of the scores. The main factor in determining sample size is this effect size. This is simply the size of the difference between treatments that is worth finding and it has been referred to as the ‘clinically relevant’ difference. It is an important point to note that the sample size obtained from equation (1) is the same for both $+d$ and for $-d$, that is, whether the patients get better or get worse with respect to HRQoL with the new treatment. In contrast, for a strongly skewed distribution, it does effect the sample size if the score is anticipated to be decreased rather than increased (Julious et al, 1995, 1997; Campbell et al, 1996).

### *Ordered categorical method*

Most HRQoL scales have categories that can be ordered, but the scores should not be treated as meaningful numbers, for example, a change in HADS from 5 to 10 is not the same as a change from 10 to 15. However, methods have been developed for sample size calculations for ordered categorical (ordinal) data (Whitehead, 1993).

Equation (2) is based on the Mann-Whitney *U*-test for ordered categorical data. It estimates the sample size based on the odds ratio (*OR*) of a patient being in a given category or less in one treatment group compared to the other group. Here $k$ is the number of categories on the HRQoL instrument, $\bar{p}_i$ is the mean proportion expected in category $i$, that is, $\bar{p}_i = (p_{Ai} + p_{Bi})/2$, where $p_{Ai}$ and $p_{Bi}$ are the proportions anticipated in category $i$ for the two treatment groups $A$ and $B$ respectively.

$$N = \frac{12(Z_{1-\alpha/2} + Z_{1-\beta})^2/(\log OR)^2}{[1 - \sum_{i=1}^{k} \bar{p}]^3} \qquad (2)$$

The anticipated effect size is expressed as an odds ratio defined as:

$$OR = \frac{p_{Ai}(1-p_{Bi})}{p_{Bi}(1-p_{Ai})}$$

This is a measure which is not immediately straightforward to interpret. Suppose in a clinical setting that with treatment $A$ there is an odds of 4:1 of a HADS Anxiety clinical case (the event of interest), then this implies that for every 5 patients on treatment we would expect 1 of them to be a clinical case. If however on $B$ the odds were lengthened to 8:1, then one would have an *OR* =(4/1)/(8/1)= 0.50 in favour of *B*. In general, an *OR* should not be interpreted as though it were a relative risk (*RR*). Using the same example, 20.0% (1/5) of patients are clinical cases on *A*, whereas 11.1% (1/9) are with *B*, giving a relative risk, *RR* = 11.1/20.0 = 0.56 in favour of *B*. This is close, but not equal, to the value of the corresponding *OR*. However, as two such event rates lower the

**Table 1** Frequency of responses on the HADS Anxiety scores at baseline for patients with small-cell lung cancer (data from Medical Research Council Lung Cancer Working Party, 1996)

| Category | Score | Number of patients |
|---|---|---|
| Normal | 0 | 0 |
| | 1 | 0 |
| | 2 | 1 |
| | 3 | 0 |
| | 4 | 2 |
| | 5 | 3 |
| | 6 | 5 |
| | 7 | 10 |
| Borderline | 8 | 12 |
| | 9 | 15 |
| | 10 | 24 |
| Clinical case | 11 | 41 |
| | 12 | 49 |
| | 13 | 36 |
| | 14 | 23 |
| | 15 | 34 |
| | 16 | 9 |
| | 17 | 2 |
| | 18 | 0 |
| | 19 | 0 |
| | 20 | 0 |
| | 21 | 0 |
| | Total | 266 |
| Normal | 0–7 | 21 (7.9%) |
| Borderline | 8–10 | 51 (19.2%) |
| Clinical case | 11–21 | 194 (72.9%) |
| Mean | | 11.70 |
| SD (σ) | | 2.66 |
| Median | | 12 |

© 2000 Cancer Research Campaign

**Table 2** Sample size estimates by the Normal distribution assumption and ordered categorical approach for a two treatment parallel group clinical trial for specified anticipated difference between treatments on the HADS Anxiety score (two-sided, α = 5% and power, 1-β = 80%)

| | Anticipated difference | | | | | |
|---|---|---|---|---|---|---|
| **Method** | **–3** | **–2** | **–1** | **+1** | **+2** | **+3** |
| Normal | 28[a] | 58 | 224 | 224 | 58 | 28 |
| Ordered categorical | 42 | 98 | 1048 | 96 | 40 | 10 |

[a] In practice all these will be rounded upwards, to 30, 60, 230, etc.

odds ratio and the relative risk become closer and closer in numerical value.

When designing a clinical trial, to estimate the odds ratio one can utilise the predefined clinical cut points that the HADS and RSCL each provide. For example, 27.1% of patients are defined non-clinical cases on the HADS Anxiety dimension score at baseline (see Table 1), that is, 27.1% record values resulting in a score of ≤10. This we will take, for planning purposes, as what we would expect on standard therapy (*S*). The odds with *S* is thus 0.271/(1 – 0.271) = 0.372. Suppose a new therapy (*T*) is to be studied and the investigator decided that a clinically meaning effect is one that would increase the proportion of non-cases by 10%, that is, from 27.1 to 37.1% or a postulated odds of 0.371/(1 – 0.371) = 0.590. The ratio of these odds gives *OR* = 0.372/0.590 = 0.63 in favour of *T*. This value can then be used as the basis for the sample size calculation.

Equation (2) makes no assumption about the distribution of the data, but it does assume proportional odds between the treatments across the HRQoL dimension. This implies that the odds ratios are identical for each pair of adjacent categories throughout the scale. What this means practically can be highlighted by extending the example given above. When using the pre-defined clinical cut point for 'non-cases' the investigator anticipated the *OR* would be 0.63. The assumption of proportional odds implies that, if instead of using ≤10 as the definition of a 'non-case', ≤9 had been used, one would nevertheless obtain $OR_9$ = 0.63; and so on for $OR_8$, $OR_7$, etc. Thus, although the actual observed odds ratios might differ from each other across the scale, the corresponding population values are all equal which implies that $OR_1 = OR_2 = OR_3 = \ldots = OR_{21} = 0.63$. However, the calculations of sample size using equation (2) are robust to departures from this ideal, provided all the odds ratios indicate an advantage to the same treatment.

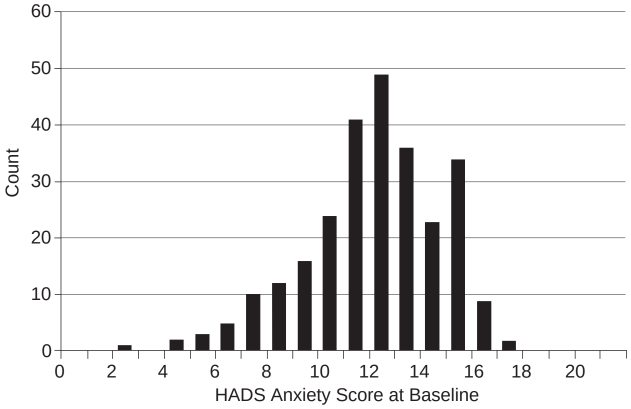

**Figure 1** Distribution of HADS anxiety scores at baseline (data from Medical Research Council Lung Cancer Working Party, 1996)

### RESULTS

### Distributions

Figure 1 displays the distribution of the HADS anxiety scores at baseline. It is negatively skewed. Figure 2 shows the equivalent distribution of the RSCL psychological dimension scores. It is positively skewed. In either case the scores do not have even approximately the Normal distribution form. It therefore seems that the usual mean and standard deviation are not adequate to summarize the distributions. As a consequence, distribution-free techniques should be used for testing treatment differences.

### Comparison of methods

For expository purposes the HADS Anxiety scores at baseline, given in Table 1, will be taken as the scores we anticipate for patients on standard therapy (*S*). We further assume that we are planning a randomized trial where we wish to demonstrate the benefit of a new therapy (*T*) against this standard.

For purposes of calculating sample sizes we make an assumption that the differences of interest range from –3 to +3 from the population mean (or median) of *S*. In each example, the sample sizes are calculated taking a two-sided significance level of 5% and 80% power.

#### *Normal distribution method*

From Table 1 the anticipated mean score for the HADS Anxiety scores for patients on *S* is 11.7 and thus a difference of 1 unit of HRQoL would be for *T* to reduce this mean score to 10.7. The anticipated standardized difference of interest is then, $d = (\mu_A - \mu_B)/\sigma = (11.7 - 10.7)/2.66 = +0.376$. Using equation (1), the required sample size is estimated as *N* = 224 patients. If however, we suspected that *T* would increase the mean HADS Anxiety score rather than decrease it, then the corresponding standardized difference becomes *d* = (11.7 – 12.7)/2.66 = –0.376. From equation (1) the sample size is again *N* = 224 patients. The results for various anticipated difference in HADS anxiety scores are summarized in the corresponding row of Table 2. It is thus evident that the methodology, which assumes a symmetric (Normal) distribution for the resulting data for the corresponding HRQoL dimension, gives symmetric sample sizes. Thus the sample size obtained depends only on the absolute value of the anticipated standardized difference between treatments.

#### *Ordered categorical method*

For ordered categorical data, it is usually more informative to describe the results in terms of the median rather than the mean.

© 2000 Cancer Research Campaign

**Table 3** Anticipated percentages of responses on the HADS Anxiety scores for standard treatment (*S*) and new treatment (*T*) for patients with small-cell lung cancer (data from Medical Research Council Lung Cancer Working Party, 1996)

| Category | Score[a] | Standard therapy (*S*) | | New therapy (*T*) | |
|---|---|---|---|---|---|
| | | Percentage ($P_{Si}$) | Cumulative percentage ($Q_{Si}$) | Percentage ($p_{Ti}$) | Cumulative percentage ($Q_{Ti}$) |
| Normal | 0–3 | 0.4 | 0.4 | 0.5 | 0.5 |
| | 4 | 0.8 | 1.2 | 1.1 | 1.6 |
| | 5 | 1.1 | 2.3 | 1.5 | 3.1 |
| | 6 | 1.9 | 4.2 | 2.5 | 5.6 |
| | 7 | 3.8 | 8.0 | 4.9 | 10.5 |
| Borderline | 8 | 4.5 | 12.5 | 5.7 | 16.2 |
| | 9 | 5.6 | 18.1 | 6.8 | 23.0 |
| | 10 | 9.0 | 27.1 | 10.0 | 33.5 |
| Clinical case | 11 | 15.4 | 42.5 | 16.5 | 50.0 |
| | 12 | 18.4 | 60.9 | 17.8 | 67.8 |
| | 13 | 13.5 | 74.4 | 11.9 | 79.7 |
| | 14 | 8.6 | 83.0 | 7.1 | 86.9 |
| | 15 | 12.8 | 95.8 | 10.0 | 96.9 |
| | 16 | 3.4 | 99.2 | 2.5 | 99.4 |
| | 17–21 | 0.8 | 100.0 | 0.6 | 100.0 |

[a] The 22 categories of Table 1 are reduced to $k = 15$.

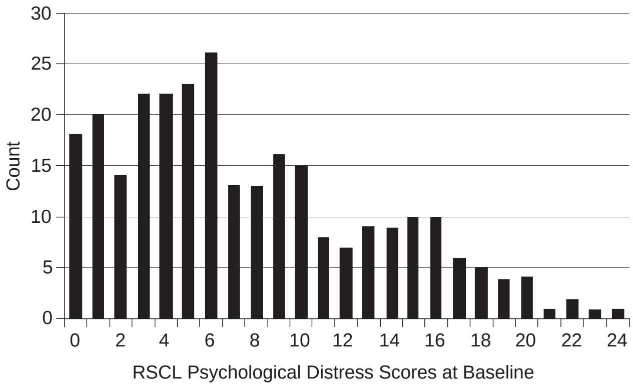

**Figure 2** Distribution of RSCL psychological distress scores at baseline (data from Medical Research Council Lung Cancer Working Party, 1996)

Thus the median score for *S* is 12 (Table 1) and sample sizes can therefore be derived for the situations where the anticipated median on *T* is either reduced or increased. The calculations in the worked example of Table 3 are for a reduction in the median score to 11.

The first two columns of Table 3 gives the proportion and cumulative proportions anticipated for each possible score of the HADS Anxiety dimension and are based on those from the data given in Table 1. Thus 60.9% of patients receiving S are anticipated in the median score category 12 or less. The median score on *T* would therefore be reduced by one unit at least, and the clinical problems eased, if half (50%) or more of the patients on that treatment fell into score category ≤11. As 42.5% of patients receiving *S* are anticipated to be ≤11, the anticipated odds ratio for sample size calculation purposes is determined as $OR = (0.500 \times 0.425)/(0.575 \times 0.500) = 0.739$.

With this odds-ratio, and the proportions anticipated on *S*, the anticipated cumulative proportions lying in each successive score cell for *T* can be derived from $Q_{Ti} = Q_{Si}/[Q_{Si} + OR\,(1 - Q_{Si})]$, where $Q_{Si}$ is the cumulative proportion in category *i* for treatment *S*. Thus, for example, the anticipated proportion for score category 10 is $Q_{T10} = Q_{S10}/[Q_{S10} + OR(1 - Q_{S10})] = 0.271/[0.271 + 0.739(1 - 0.271)] = 0.335$. Similarly, the cumulative proportions can be calculated for the other categories and, from these, the anticipated proportions derived and the final two columns in Table 3 completed. The mean proportion for each of the $k = 15$ categories can now be estimated by: $\bar{p}_{0-3} = (0.004 + 0.005)/2 = 0.005$, $\bar{p}_4 = (0.008 + 0.011)/2 = 0.010$, $\bar{p}_5 = 0.013$, $\bar{p}_6 = 0.022$, …, $\bar{p}_{17-21} = 0.007$. The sample size can now be calculated using equation (2), which gives $N = 1048$.

These calculations were applied to the range of differences and the results are summarized in the final row of Table 2. It is therefore evident that using equation (2) leads to asymmetric sample sizes: the size depending on the sign of the difference.

The application of proportional odds therefore allows that, if the distribution of one of the treatment groups can be specified, then the anticipated cumulative proportions for the other treatment can be directly derived. Hence, with prior knowledge of the distribution of just one treatment group and an anticipated *OR*, obtained about any cut point on the HRQoL scale, an estimate of the sample size can be obtained.

### Number of categories

Despite the presence of a full ordered categorical scale, researchers often estimate sample size and analyse studies, using an odds ratio determined from a pre-defined score determining a case and thereby ignore the other points on the HRQoL scale. For example, with the HADS Anxiety dimension they simply classify subjects as either a case or non-case. In this now binary data situation, equation (2) can still be used to estimate sample sizes but ignoring the full ordered categorical nature of the data, may result in a substantial over-estimate of the necessary trial size. For example, if a clinically meaningful difference was set as an increase in the number of subjects that are non-cases on the HADS Anxiety score from 27.1% to 40.0% then this equates to an

 *© 2000 Cancer Research Campaign*

$OR = (0.271/0.729)/(0.400/0.600) = 0.58$. Using this in equation (2) gives $N_2 = 414$ compared to only 282 using all $k = 15$ categories in the calculations. This is an over-estimate of 47% in the necessary sample size.

However, it may not be essential to use the full categorical scale. For example, with HADS there is an additional category of ‘normal’ for subjects with a score of ≤8 and just under 8% of patients are classified as such on the anxiety dimension (Table 2 and 3). If one then calculated the sample size using the $k = 3$ groups of ‘normal’, ‘borderline-case’ and ‘clinical-case’ as the categories the estimated sample size is $N_3 = 400$ subjects – only a marginally closer estimate. However, if one identified an additional category of ‘severe-clinical-case’ for subjects with a HADS score ≥14 and based the sample size calculations on the 4 categories, the estimated sample size is $N_4 = 310$ which is now quite close to the optimal 282. Thus, only a modest increase in the complexity of the calculations can lead to substantially better estimates – choosing not more than $k = 5$ categories is usually sufficient.

### Choosing an effect size

Probably the most important component in the estimation of the sample size is the effect size. If one halves this one quadruples the sample size (Fayers and Machin, 1995). However, for HRQoL measures this is often the component in the calculations which one finds the most difficult to determine. Usually one can make an intelligent guess at treatment difference from clinical experience and from previously published work. However this experience has yet to be gained for much HRQoL work in many contexts (see however Fayers and Machin, 2000).

An advantage of the HADS and RSCL instruments for the process of anticipating the effect size is that they both have predefined definitions of what constitutes a ‘case’ and which can then be used to obtain a value of a readily interpretable effect size. This effect size, here expressed as an odds ratio, can thus be extended across the full HRQoL scale and an estimate of the sample size made.

### DISCUSSION

The scores resulting from the two questionnaires highlighted clearly do not have a normal distribution form. We have shown that asymmetric distributions require different sample size estimates depending on the direction of the effect size. Thus, as many HRQoL measures do not take a Normal form, the sample size estimates depend on the sign of $d$, and it is not appropriate to estimate sample sizes under the Normal assumptions of equation (1).

Further the assumption of Normality can lead to unrealistically sized trials which can be either under or over estimates of the size actually required. Our recommendation is to use the distribution-free equation (2) for sample size estimation when involving HRQoL as an outcome measure in clinical trials.

Dichotomizing the HRQoL scale in order to estimate a sample size (and consequently to analyse the subsequent data in the same way) should be avoided if possible as sample sizes could be unnecessarily inflated. However, knowledge of anticipated responses in only a handful of categories can give sample size estimates that are more precise for only a modest increase in the complexity of the calculations. We recommend therefore that when estimating sample sizes associated with the use of HRQoL instruments in clinical trials, the methods we have described should be used.

We also recommend that when reporting normative data for HRQoL scores in different populations that the full frequency distributions are given of the different dimensions. This information would greatly facilitate the planning of future clinical trials.

### REFERENCES

Campbell MJ, Julious SA and Altman DG (1995) Estimating sample size for binary, ordered categorical, and continuous outcomes in two group comparisons. *Br Med J* **311**: 1145–1148

Campbell MJ, Julious SA and George SL (1996) Estimating sample sizes for studies using the SF-36 health survey (reply to letter). *Epidemiol Comm Health* **50**: 473–474

Campbell MJ, Walker SJ, George SL, Machin D and Julious SA (2000) A review of the use of the main quality of life measures, and sample size determination for quality of life measures, particularly in cancer trials. In: *Advanced Handbook in Evidence Based Healthcare*, Steven A, Abrams KR, Brazier J, Fitzpatrick R and Lilford RJ (eds) Sage Publications: London de Haes JCJM and van Knippenberg FCE (1985) The quality of life of cancer patients – a review of the literature. *Soc Sc Med* **20**: 809–817

de Haes JCJM, van Knippenberg FCE and Neijt JP (1990) Measuring psychological and physical distress in cancer patients: structure and application of the Rotterdam Symptom Checklist. *Br J Cancer* **62**: 1034–1038

Fayers PM and Machin D (1995) Sample size – how many patients are necessary? *Br J Cancer* **72**: 1–9

Fayers PM and Machin D (2000) *Quality of Life: Assessment, Analysis and Interpretation*. John Wiley: Chichester

Julious SA and Campbell MJ (1996) Sample size calculations for ordered categorical data. *Stats in Med* **15**: 1065–1066

Julious SA, George S and Campbell MJ (1995) Sample size for studies using the short form 36 (SF-36). *J Epidemiol Comm Health* **49**: 642–644

Julious SA, George SL, Machin D and Stephens RJ (1997) Sample sizes for randomised trials measuring quality of life in cancer patients. *Quality of Life Research* **6**: 109–117

Machin D, Campbell MJ, Fayers PM and Pinol APY (1997) *Statistical Tables for the Design of Clinical Studies*. Blackwell Scientific: Oxford

Medical Research Council Lung Cancer Working Party (1996) Randomised trial of four-drug vs less intensive two-drug chemotherapy in the palliative treatment of patients with small-cell lung cancer (SCLC) and poor prognosis. *Br J Cancer* **73**: 406–413

Moorey S, Greer S, Watson M, Gorman C, Rowden L, Tunmore R, Robertson B and Bliss J (1991) The factor structure and factor stability of the Hospital Anxiety and Depression Scale in patients with cancer. *Br J Psych* **158**: 255–259

Whitehead J (1993) Sample size calculations for ordered categorical data. *Stats in Med* **12**: 2257–2273

Zigmond AS and Snaith RP (1983) The Hospital Anxiety and Depression Scale. *Acta Psychiatric Scand* **67**: 361–370

© 2000 Cancer Research Campaign
